# EQ-5D-3L Health Status Among Homeless People in Stockholm, Sweden, 2006 and 2018

**DOI:** 10.3389/fpubh.2021.780753

**Published:** 2021-12-20

**Authors:** Bo Burström, Robert Irestig, Kristina Burström

**Affiliations:** ^1^Equity and Health Policy Research Group, Department of Global Public Health, Karolinska Institutet, Stockholm, Sweden; ^2^Capio Psychiatry, Stockholm, Sweden; ^3^Health Outcomes and Economic Evaluation Research Group, Department of Learning, Informatics, Management and Ethics, Stockholm Centre for Healthcare Ethics, Karolinska Institutet, Stockholm, Sweden

**Keywords:** EQ-5D-3L, health-related quality of life, homeless people, inequalities in health, interviews, social determinants of health

## Abstract

**Background:** Homeless people are a socially excluded group whose health reflects exposures to intersecting social determinants of health. The aim of this study was to describe and compare the demographic composition, certain social determinants of health, and self-reported health among homeless people in Stockholm, Sweden, in 2006 and 2018.

**Methods:** Analysis of data from face-to-face interviews with homeless people in Stockholm 2006 (*n* = 155) and 2018 (*n* = 148), based on a public health survey questionnaire adapted to the group, including the EQ-5D-3L instrument. The chi-squared test was employed to test for statistical significance between groups and the independent *t*-test for comparison of mean scores and values. Ordinary Least Squares (OLS) regression, with Robust Standard Errors (RSE) was performed on merged 2006 and 2018 data with mean observed EQ VAS score as outcome variable.

**Results:** In 2018 more homeless people originated from countries outside Europe, had temporary social assistance than long-term social insurance, compared to in 2006. In 2018 more respondents reported lack of social support, exposure to violence, and refrained from seeking health care because of economic reasons. Daily smoking, binge drinking, and use of narcotic drugs was lower 2018 than 2006. In 2018 a higher proportion reported problems in the EQ-5D-3L dimensions, the mean TTO index value and the VAS index value was significantly lower than in 2006. In the regression analysis of merged data there was no significant difference between the years.

**Conclusions:** Homeless people are an extremely disadvantaged group, have high rates of illness and disease and report poor health in all EQ-5D-3L dimensions. The EQ VAS score among the homeless people in 2018 is comparable to the score among persons aged 95–104 years in the general Swedish population 2017. The EQ-5D-3L instrument was easily administered to this group, its use allows comparison with larger population groups. Efforts are needed regarding housing, but also intensified collaboration by public authorities with responsibilities for homeless people's health and social welfare. Further studies should evaluate the impact of such efforts by health and social care services on the health and well-being of homeless people.

## Introduction

Homeless people are a socially excluded group with poor mental and physical health, partly resulting from exposures to intersecting social determinants of health (1–4). The composition of the group of homeless people and their health is also related to how the surrounding society is organized (3), which job and housing opportunities are available, and which health and social welfare services are in place. Recently, the COVID-19 pandemic has also demonstrated the increased overall vulnerability of homeless people, both to exposure to the virus, to the severity and impact of the disease and their vulnerability to the consequences of the disease (5, 6).

Homeless people have higher rates of mental disorders and addiction, as well as somatic diseases. Their mortality risk is several times higher than that in the general population (4). A high proportion of homeless people have grown up under adverse circumstances; some have mental health problems which they sometimes self-medicate with narcotic drugs (3). A study in Stockholm found that excess mortality among homeless people was strongly related to alcohol and drug abuse (7). As a group, homeless people tend to acquire somatic diseases at an earlier age than the general population, and thereby also have an increased risk of getting complications, for instance of developing cardiovascular disease as a consequence of having diabetes mellitus (3). The impact of the social determinants of health is particularly evident in the group (8). The hardship of daily life circumstances of homeless people, not having a steady place to sleep and be safe, lacking money and social support, and constantly looking for food and shelter, further exacerbates their mental and physical health status (9). Being homeless is associated with increased risk of violence, assaults, and injuries are common, homeless women are subjected to sexual violence, a high proportion of homeless people use narcotic drugs which in turn may be linked to criminal activities (10). Homeless people are also frequently in contact with public authorities such as health care services (11), social services, and the police, and their trust in public institutions is lower compared to the general public (1). The health of homeless people is evidently impacted by social determinants of health (9) and represents an extreme of social inequalities in health (8).

In comparison with many other countries, Sweden has a comprehensive and generous welfare system, both in terms of universal health coverage, good access to health and social services, and in terms of economic security (12). However, in the latest decades there have been restrictive changes to the Swedish social insurance system, limiting for instance the likelihood of being granted disability pension (13). The Swedish National Public Health Policy (14) focuses on the social determinants of health (15) and aims to reduce inequalities in health.

In Sweden, municipalities are obliged to plan for housing for their inhabitants, but there is no legislated right to housing and no social housing (16). However, housing has become increasingly difficult to obtain, especially for low income earners and persons with a previous history of eviction (17). The housing situation is particularly difficult in larger cities, with poverty, a shortage of affordable housing, and increasing requirements by landlords causing what in the last decade has been called “structural homelessness” (16). While municipal social services should plan housing for their inhabitants, they claim that they are only responsible for those with additional social problems (16). In addition, there has been a large influx of migrants to Sweden. In Sweden, municipalities are responsible for providing social services and social assistance to its citizens, the regions for providing health care services. In some larger cities, there are special health care services to cater for homeless people. In addition, many non-governmental organizations support homeless people with shelter, clothing, and food.

There are different degrees of homelessness. The Swedish National Board of Health and Welfare distinguish four groups (2, 17). National counts of homeless people are done every 4–5 years, using an indirect approach of surveying officials in health and social care and other organizations who work with homeless people. The latest national count in 2017, found that the number of homeless people had increased to 33,400 from 30,800 in 2011 (17). The increase was greatest (32%) in the group referred to as “acute homeless” (rough sleepers, people sleeping in shelters, emergency housing, and protected housing), from 4,500 to 5,935 persons. The city (municipality) of Stockholm also performs similar counts, locally. In 2018, the number of homeless people was 2,439, of whom 45% were considered to have obvious mental health problems and 55% had addiction problems (18).

Homeless people are seldom included in population health surveys, partly because they are difficult to reach and if reached, less possibility respond to a postal survey. Nevertheless, it is important to monitor their health and living circumstances, in order to improve the design of interventions. In 2006, a face-to-face interview survey was done with 155 homeless people in Stockholm, based on the survey questionnaire, including the generic health-related quality of life instrument EQ-5D-3L, which had been employed in a population survey to 50,000 respondents in Stockholm County the same year (2). Homelessness in that survey referred to “acute homelessness.” The publication comparing health-related quality of life of homeless people to that of the general population showed that homeless people had substantially worse health than the general population and that among the homeless, most problems were reported with anxiety and depression (2). The rates of reporting limiting longstanding illness (LLI) and less than good self-rated health (SRH) were three times higher among homeless people than in the general population. The study further found that a high proportion of homeless people reported lack of practical help, compared to the general population sample. The proportion having been exposed to violence in the past 12 months was ten times higher among homeless people (2).

A similar interview survey was done among homeless people in 2018, using the same questionnaire and mode of administration as in 2006, this time with 148 respondents.

The aim of the present study was to describe and compare the demographic composition, certain social determinants of health and self-reported health among homeless people in Stockholm, Sweden, in 2006 and 2018.

## Materials and Methods

### Participants and Settings

We used data from two face-to-face interview surveys carried out in Stockholm, Sweden, in 2006 and in 2018. At both times, the interviews took place at special lodging houses, shelters for the night, institutions, and an outpatient clinic for homeless people. In each survey, one interviewer performed all the interviews, except for two interviews in 2006 done by another interviewer. The interviewers invited potential participants who did not seem to be too intoxicated or too ill to participate. A random procedure was not possible. Approximately twice as many persons were approached as the number participating in the interviews. Among those approached, some were in a poor condition and not being able to participate or did not want to for other reasons. The interviews varied in time: from 30 min to 6 h. In 2006, two respondents had missing response on all EQ-5D-3L dimensions, hence the EQ-5D-3L analyses was based on 153 persons.

### Ethical Approval and Consent

Ethical approval was granted for both surveys by the Regional Ethical Review Board, Stockholm, Sweden (Dnr: 2006/5:7; 2018/1923-31). Prior to the interview, potential participants were informed about the purpose of the survey, that participation in the survey was voluntary and anonymous, that they were free to leave at any time. No information on name or personal identification number was collected. Verbal informed consent was obtained from each participant prior to the interview in accordance with the ethical standards of the Regional Ethical Review Board.

### Interview Questionnaire

The interview questionnaire, which was identical in both these two surveys, was based on a questionnaire used in the Public Health Survey in Stockholm County 2006. The questionnaire included demographic information (sex, age, country of origin) and other social determinants of health (level of education, occupation, social support, exposure to violence, refraining from seeking care for economic reasons, health-related behaviors). To assess self-reported health, a question on LLI, the single-item SRH question, and the EQ-5D-3L instrument were included.

That questionnaire was adapted for the interviews among homeless by harmonizing some questions to better mirror the situation for the group of homeless. For example, questions were added regarding the degree and duration of homelessness, where the respondent slept last night (to identify rough sleepers), and of different means of subsistence (including social insurance benefits or social assistance). The questionnaire used in the surveys among homeless people is described in detail in Table 1 in Sun et al. (2). In addition to the structured questions, open questions were asked about the current situation and what would help to improve.

### Limiting Longstanding Illness

Limiting longstanding illness was based on the answer to the following question: “Do you experience reduced capacity to handle work or daily activities due to any long-term illness, after-effects of accidents, disability or other ailment?”

### The Self-Rated Health Question

The single item SRH question, frequently used in surveys to assess respondents' self-reported health (19) was phrased “In your opinion, how is your health status? Is it very good, good, fair, bad, very bad?” Those who answered fair, bad or very bad were categorized as having less than good SRH.

### The EQ-5D-3L Instrument

The health-related quality of life instrument EQ-5D-3L consists of two parts: the descriptive system and a visual analog scale (VAS) the EQ VAS (20). On the first part, respondents classify their health into five dimensions (mobility, self-care, usual activities, pain/discomfort, and anxiety/depression) and three severity levels (no, moderate, and severe problems), resulting in 243 health states (3^5^). On the EQ VAS, respondents rate their own overall health between 100 (best imaginable health) and 0 (worst imaginable health), yielding an observed EQ VAS score. A single index value can be assigned to each of these 243 unique health states using a value set based on different sources of valuation and different valuation method [e.g., time trade-off (TTO) and VAS valuations]. We employed the EQ-5D-3L value sets for Sweden based on TTO and VAS valuations (21).

### Data Analysis

Descriptive analyses of the demographic composition and social determinants of health and self-reported health were calculated as proportions (percentage and numbers). Descriptive analyses of homeless people's health, as measured by the EQ-5D-3L instrument, were stratified by sex and by age, divided into four age groups (<35, 35–44, 45–54, 55+) based on the age distribution among the homeless. Proportion of respondents reporting no, moderate, and severe problems on the EQ-5D-3L dimensions, mean and median observed EQ VAS score, and mean TTO and VAS index values were calculated with standard deviation (SD) for mean values and interquartile range (IQR) for median values. The chi-squared test was employed to test for statistical significance between groups and the independent *t*-test for comparison of mean scores and values. We calculated descriptive EQ-5D-3L results by distribution of response options to the SRH question.

Ordinary least squares (OLS) regression, with Robust standard errors (RSE) was performed on merged 2006 and 2018 data with mean observed EQ VAS score as outcome variable and co-variates following Sun et al. (2), sex, age group, country of origin, duration of homelessness, degree of homelessness, health-related behaviors, LLI, and survey year (all entered as dummy variables). We used the mean observed EQ VAS score in the regression analysis as we wanted to employ the respondents own rating of their overall health perception as the outcome variable in the regression.

A 5% significance level was employed. Analyses were done in SAS software 9.4.

## Results

### Demographic Composition

A total of 148 persons (35 women and 113 men) were interviewed in 2018 and 155 persons (123 men and 32 women) in 2006 (Table 1). Women constituted 23.6% of the sample in 2018 and 20.6% in 2006. The mean age was 50.3 years in 2018, compared to 48.4 years in 2006. A majority of respondents were born in Sweden in both surveys. While persons originating from other Nordic countries was the second largest group in 2006, this proportion was significantly smaller in 2018 and a significantly greater proportion came from countries outside Europe.

**Table 1:** Demographic composition, certain social determinants of health, limiting longstanding illness, and less than good self-rated health among homeless people in 2006 and 2018, Stockholm.

	**2006 (*n* = 155)**	**2018 (*n* = 148)**	***p*-values for difference between survey years**
**Mean age** (years) [SD]	48.4 [10.3]	50.3 [2.4]	
	**% (** ***n*** **)**	**% (** ***n*** **)**	
**Sex**			
Women	20.6 (32)	23.6 (35)	0.529
Men	79.4 (123)	76.4 (113)	
**Country of origin**			
Sweden	68.1 (105)	66.0 (97)	0.685
Other Nordic countries	13.6 (21)	6.1 (9)	0.030
Other European countries	4.6 (7)	4.1 (6)	0.843
Other countries outside Europe	13.6 (21)	23.8 (35)	0.024
**Level of education**			
Primary school 9 years or less	57.1 (88)	51.7 (76)	0.344
Secondary school 2–3 years	37.4 (58)	37.1 (55)	0.963
University	5.2 (8)	10.8 (16)	0.069
**Duration of homelessness**			
<4 years	26.4 (41)	20.3 (30)	0.204
4–9 years	29.7 (46)	29.1 (43)	0.905
10+ years	41.9 (65)	50.7 (75)	0.127
**Degree of homelessness**			
Rough sleepers	11.8 (18)	10.8 (16)	0.825
**Subsistence**			
Social insurance	47.7 (74)	18.9 (28)	<0.0001
Receiving social assistance	37.4 (58)	52.7 (78)	0.008
**Social relations**			
Lack practical help	25.2 (39)	50.0 (74)	<0.0001
**Violence**			
Exposed to physical violence	31.0 (48)	40.5 (60)	0.082
**Care and economic situation**			
Refraining from seeking dental care	67.7 (105)	66.9 (99)	0.875
Refraining from seeking health care	31.6 (49)	48.0 (71)	0.004
Refraining from purchasing prescribed drugs	25.8 (40)	48.6 (72)	<0.001
**Health-related behaviors**			
Smoking daily	80.6 (125)	68.2 (101)	0.013
Binge drinking	24.5 (38)	20.3 (30)	0.187
Narcotic drug use	50.0 (76)	35.1 (52)	0.014
**Self-reported health**			
Limiting longstanding illness	61.3 (95)	71.6 (106)	0.057
Less than good self-rated health	63.4 (97)	77.7 (115)	0.004

### Social Determinants of Health

Most respondents had only mandatory school (9 years) or less in both surveys; <40% had secondary school (Table 1). The proportion with university education was higher in 2018. In 2018, a higher proportion (50.7%) of the interviewees reported to have been homeless for 10 years or longer, than in 2006 (41.9%). The proportion of rough sleepers the night before the interview was similar.

The proportion reporting social insurance as main subsistence in 2018 was 18.9%, significantly lower than in 2006 (47.7%). In 2018, a significantly higher proportion reported receiving social assistance (52.7%) than in 2006 (37.4%). The proportions reporting other sources of income (work, unemployment benefit, pension, criminal activities) were similar in the two surveys (data not shown).

Regarding social support, in 2018, a significantly higher proportion (50%) of respondents reported lacking practical help, compared to 25.4% in 2006. The proportion reporting having been exposed to violence in the last 12 months was significantly higher in 2018 than in 2006. In both surveys two-thirds of respondents reported to have refrained from seeking dental care for economic reasons, while the proportions refraining from seeking health care and purchasing prescribed drugs were significantly higher in 2018 than in 2006.

The proportion reporting daily smoking was significantly lower in 2018 (68.2%) than in 2006 (80.6%), binge drinking was lower in 2018 (20.3%) than in 2006 (24.5%), and the proportion reporting to use narcotic drugs was significantly lower in 2018 (35.1%) compared to 2006 (50.0%) (Table 1).

### Limiting Longstanding Illness and Self-Rated Health

The proportions reporting to have a LLI and to have less than good SRH were high in both surveys, significantly higher in 2018 than in 2006 for SRH (Table 1).

### Homeless People's Health-Related Quality of Life

In 2018, the proportion of respondents reporting moderate or severe problems was higher than in 2006 in most EQ-5D-3L dimensions, significantly so in the dimensions usual activities and pain/discomfort, especially in the age group 45–54 years, but also in the age group 35–44 years for pain/discomfort (Table 2). The proportion reporting severe problems was higher in 2018 than in 2006 in all dimensions. The mean EQ VAS score was lower in 2018 than in 2006, but the difference was not significant. The mean TTO and VAS index values in the total samples were significantly lower in 2018 than in 2006, and the difference was significant in the age group 45–54 years (Table 2).

**Table 2:** Problems in EQ-5D-3L dimensions (%, *n*), mean and median EQ VAS score, mean TTO index value, mean VAS index value, by age group, homeless people in 2006 and 2018, Stockholm.

**EQ-5D-3L dimensions**	**Age groups (years)**									
	**Total**		** <35**		**35–44**		**45–54**		**55+**	
	**2006**	**2018**	**2006**	**2018**	**2006**	**2018**	**2006**	**2018**	**2006**	**2018**
	**% (*n*)**	**% (*n*)**	**% (*n*)**	**% (*n*)**	**% (*n*)**	**% (*n*)**	**% (*n*)**	**% (*n*)**	**% (*n*)**	**% (*n*)**
**Mobility**										
No problems	73.9 (113)	61.5 (91)	100.0 (11)	84.2 (16)	85.0 (34)	88.0 (22)	71.2 (42)	52.1 (25)	60.5 (26)	50.0 (28)
Moderate problems	25.5 (39)	37.2 (55)	0.0 (0)	15.8 (3)	15.0 (6)	12.0 (3)	27.1 (16)	45.8 (22)	39.5 (17)	48.2 (27)
Severe problems	0.6 (1)	1.4 (2)	0.0 (0)	0.0 (0)	0.0 (0)	0.0 (0)	1.7 (1)	2.1 (1)	0.0 (0)	1.8 (1)
**Self-Care**										
No problems	93.5 (143)	91.2 (135)	100.0 (11)	100.0 (19)	97.5 (39)	96.0 (24)	96.6 (57)	89.6 (43)	83.7 (36)	87.5 (49)
Moderate problems	6.5 (10)	7.4 (11)	0.0 (0)	0.0 (0)	2.5 (1)	0.0 (0)	3.4 (2)	8.3 (4)	16.3 (7)	12.5 (7)
Severe problems	0.0 (0)	1.4 (2)	0.0 (0)	0.0 (0)	0.0 (0)	4.0 (1)	0.0 (0)	2.1 (1)	0.0 (0)	0.0 (0)
**Usual Activities**										
No problems	79.7 (122)	60.8 (90)^*^	81.8 (9)	52.6 (10)	77.5 (31)	68.0 (17)	84.8 (50)	62.5 (30)^*^	74.4 (32)	58.9 (33)
Moderate problems	17.7 (27)	31.1 (46)	18.2 (2)	36.8 (7)	20.0 (8)	28.0 (7)	15.2 (9)	27.1 (13)	18.6 (8)	33.9 (19)
Severe problems	2.6 (4)	8.1 (12)	0.0 (0)	10.5 (0)	2.5 (1)	4.0 (1)	0.0 (0)	10.4 (5)	7.0 (3)	7.1 (4)
**Pain/Discomfort**										
No problems	42.1 (64)	21.6 (32)^*^	54.6 (6)	42.1 (8)	61.5 (24)	24.0 (6)^*^	35.6 (21)	14.6 (7)^*^	30.2 (13)	19.6 (11)
Moderate problems	41.5 (63)	48.7 (72)	36.4 (4)	47.4 (9)	23.1 (9)	60.0 (15)	49.2 (29)	39.6 (19)	48.8 (21)	51.8 (29)
Severe problems	16.5 (25)	29.7 (44)	9.1 (1)	10.5 (2)	15.4 (6)	16.0 (4)	15.2 (9)	45.8 (22)	20.9 (9)	28.6 (16)
**Anxiety/Depression**										
No problems	26.7 (40)	20.3 (30)	18.2 (2)	21.1 (4)	28.2 (11)	16.0 (4)	22.4 (13)	25.0 (12)	33.3 (14)	17.9 (10)
Moderate problems	47.3 (71)	42.6 (63)	45.4 (5)	21.1 (4)	48.7 (19)	52.0 (13)	51.7 (30)	37.5 (18)	40.5 (17)	50.0 (28)
Severe problems	26.0 (39)	37.2 (55)	36.4 (4)	57.9 (11)	23.1 (11)	32.0 (8)	25.9 (15)	37.5 (18)	26.2 (11)	32.1 (18)
**EQ VAS Score (mean)**	56.5	53.4	49.6	57.9	61.3	52.6	57.6	51.8	52.4	53.5
**[SD]**	[23.4]	[26.7]	[25.5]	[37.7]	[21.4]	[25.5]	[22.7]	[27.0]	[25.1]	[23.7]
**EQ VAS score (median)**	60.0	50.0	50.0	55.0	40.0	60.0	62.5	50.0	50.0	50.0
**[IQR]**	[35.0]	[40.0]	[35.0]	[82.0]	[40.0]	[45.0]	[25.0]	[38.0]	[40.0]	[37.5]
**TTO index value (mean)**	0.808	0.730^*^	0.827	0.724	0.830	0.775	0.807	0.711^*^	0.784	0.729
**[SD]**	[0.150]	[0.156]	[0.144]	[0.168]	[0.142]	[0.134]	[0.138]	[0.173]	[0.173]	[0.144]
**VAS index value (mean)**	65.3	54.8^*^	68.3	55.4	68.5	60.3	64.6	52.1^*^	62.3	54.4
**[SD]**	[19.5]	[19.7]	[19.1]	[21.9]	[18.9]	[17.4]	[17.9]	[21.6]	[22.1]	[18.2]

*Moderate and severe problems were collapsed into any problems when testing for significant differences*.

* *Statistically significant difference (<0.05) between survey years*.

Sex differences in HRQoL are shown in Table 3. Although women in the 2018 survey generally reported worse health and more problems than in 2006, the differences were not statistically significant. Men in the 2018 survey reported more problems and more severe problems in all dimensions than men in the 2006 survey; the differences were statistically significant in the dimensions usual activities and pain/discomfort. The mean EQ VAS score was lower in 2018 than in 2006, both among men and women, but the difference was not significant. The mean TTO and VAS index values were lower among both men and women in 2018 than in 2006; significantly lower among men (Table 3).

**Table 3:** Problems in EQ-5D-3L dimensions (%, *n*), mean and median EQ VAS score, mean TTO index value, mean VAS index value, by sex, homeless people in 2006 and 2018, Stockholm.

**EQ-5D-3L dimensions**	**Men**		**Women**	
	**2006**	**2018**	**2006**	**2018**
	**% (*n*)**	**% (*n*)**	**% (*n*)**	**% (*n*)**
**Mobility**				
No problems	72.3 (88)	59.3 (67)	78.1 (25)	68.6 (24)
Moderate problems	26.5 (32)	39.8 (45)	21.9 (7)	28.6 (10)
Severe problems	0.8 (1)	0.9 (1)	0.0 (0)	2.9 (1)
**Self-Care**				
No problems	91.7 (111)	90.3 (102)	100.0 (32)	94.3 (33)
Moderate problems	8.3 (10)	8.0 (9)	0.0 (0)	5.7 (2)
Severe problems	0.0 (0)	1.8 (2)	0.0 (0)	0.0 (0)
**Usual Activities**				
No problems	82.6 (100)^*^	62.8 (71)	68.8 (22)	54.3 (19)
Moderate problems	15.7 (19)	31.0 (35)	25.0 (8)	31.4 (11)
Severe problems	1.7 (2)	6.2 (7)	6.2 (2)	14.3 (5)
**Pain/Discomfort**				
No problems	41.3 (50)^*^	17.7 (20)	45.2 (14)	34.3 (12)
Moderate problems	42.2 (51)	52.2 (59)	38.7 (12)	37.1 (13)
Severe problems	16.5 (20)	30.1 (34)	16.1 (5)	28.6 (10)
**Anxiety/depression**				
No problems	28.8 (34)	20.4 (23)	18.8 (6)	20.0 (7)
Moderate problems	46.6 (55)	45.1 (51)	50.0 (16)	34.3 (12)
Severe problems	24.6 (29)	34.5 (39)	31.2 (10)	45.7 (16)
**EQ VAS score (mean)**	54.9	52.5	62.6	56.2
**[SD]**	[23.9]	[25.6]	[20.6]	[29.9]
**EQ VAS score (median)**	55.0	50.0	62.5	60.0
**[IQR]**	[35.0]	[40.0]	[35.0]	[50.0]
**TTO index value (mean)**	0.814	0.735^*^	0.787	0.716
**[SD]**	[0.146]	[0.153]	[0.165]	[0.166]
**VAS index value (mean)**	66.0	55.1^*^	62.6	53.7
**[SD]**	[19.0]	[19.3]	[21.3]	[21.2]

*Moderate and severe problems were collapsed into any problems when testing for significant differences*.

* *Statistically significant difference (<0.05) between survey years*.

Of the possible 243 EQ-5D-3L health states, 48 were reported in 2018 and 36 were reported in 2006 (Supplementary Material).

In both surveys, there was in general a gradient by SRH severity levels in the reporting of moderate and severe problems in different EQ-5D-3L dimensions, with more problems reported by those with worse SRH (Table 4). Respondents reporting very bad SRH had the lowest mean EQ VAS scores and lowest mean TTO and VAS index values.

**Table 4:** Problems in EQ-5D-3L dimensions (%, *n*), mean and median EQ VAS score, mean TTO index value, mean VAS index value, by self-rated health (SRH) severity level, homeless people in 2006 and 2018, Stockholm.

**EQ-5D-3L dimensions**	**SRH severity levels**									
	**Very good**		**Good**		**Fair**		**Bad**		**Very bad**	
	**2006**	**2018**	**2006**	**2018**	**2006**	**2018**	**2006**	**2018**	**2006**	**2018**
	**% (*n*)**	**% (*n*)**	**% (*n*)**	**% (*n*)**	**% (*n*)**	**% (*n*)**	**% (*n*)**	**% (*n*)**	**% (*n*)**	**% (*n*)**
**Mobility**										
No problems	94.4 (17)	63.6 (7)	94.7 (36)	86.4 (19)	73.1 (38)	71.2 (37)	48.4 (15)	52.5 (21)	50.0 (7)	30.4 (7)
Moderate problems	5.6 (1)	36.4 (4)	5.3 (2)	13.6 (3)	26.9 (14)	28.8 (15)	48.4 (15)	47.5 (19)	50.0 (7)	60.9 (14)
Severe problems	0.0 (0)	0.0 (0)	0.0 (0)	0.0 (0)	0.0 (0)	0.0 (0)	3.2 (1)	0.0 (0)	0.0 (0)	8.7 (2)
**Self-Care**										
No problems	100.0 (18)	90.9 (10)	100.0 (38)	95.4 (21)	94.2 (49)	96.2 (50)	90.3 (28)	90.0 (36)	71.4 (10)	78.3 (18)
Moderate problems	0.0 (0)	9.1 (1)	0.0 (0)	4.6 (1)	5.8 (3)	3.8 (2)	9.7 (3)	10.0 (4)	28.6 (4)	13.0 (3)
Severe problems	0.0 (0)	0.0 (0)	0.0 (0)	0.0 (0)	0.0 (0)	0.0 (0)	0.0 (0)	0.0 (0)	0.0 (0)	8.7 (2)
**Usual Activities**										
No problems	100.0 (18)	100.0 (11)	86.8 (33)	86.4 (19)	78.9 (41)	65.4 (34)	71.0 (22)	57.5 (23)	57.1 (8)	13.0 (3)
Moderate problems	0.0 (0)	0.0 (0)	13.2 (5)	13.6 (3)	19.2 (10)	30.8 (16)	25.8 (8)	35.0 (14)	28.6 (4)	56.5 (13)
Severe problems	0.0 (0)	0.0 (0)	0.0 (0)	0.0 (0)	1.9 (1)	3.8 (2)	3.2 (1)	7.5 (3)	14.3 (2)	30.4 (7)
**Pain/Discomfort**										
No problems	72.2 (13)	45.4 (5)	70.3 (26)	40.9 (9)	36.5 (19)	21.2 (11)	12.9 (4)	17.5 (7)	14.3 (2)	0.0 (0)
Moderate problems	22.2 (4)	36.4 (4)	24.3 (9)	45.5 (10)	48.1 (25)	50.0 (26)	64.5 (20)	57.5 (23)	35.7 (5)	39.1 (9)
Severe problems	6.6 (1)	18.2 (2)	5.4 (2)	13.6 (3)	15.4 (8)	21.8 (15)	22.6 (7)	25.0 (10)	50.0 (7)	60.9 (14)
**Anxiety/Depression**										
No problems	66.7 (12)	63.6 (7)	36.8 (14)	31.8 (7)	14.0 (7)	21.1 (11)	16.7 (5)	2.5 (1)	14.3 (2)	17.4 (4)
Moderate problems	33.3 (6)	27.3 (3)	57.9 (22)	63.6 (14)	62.0 (31)	51.9 (27)	30.0 (9)	40.0 (16)	21.4 (3)	13.0 (3)
Severe problems	0.0 (0)	9.1 (1)	5.3 (2)	4.6 (1)	24.0 (12)	26.9 (14)	53.3 (16)	57.5 (23)	64.3 (9)	69.6 (16)
**EQ VAS score (mean)**	84.2	90.3	68.3	72.4	54.6	56.8	42.2	42.3	28.6	28.9
**[SD]**	[13.3]	[10.0]	[18.7]	[17.1]	[18.1]	[20.7]	[17.9]	[20.5]	[18.6]	[27.8]
**EQ VAS score (median)**	80.0	90.0	75.0	75.0	50.0	55.0	40.0	40.0	25.0	20.0
**[IQR]**	[27.0]	[20.0]	[20.0]	[25.0]	[30.0]	[27.5]	[20.0]	[20.0]	[35.0]	[40.0]
**TTO index value (mean)**	0.932	0.868	0.892	0.865	0.802	0.765	0.708	0.675	0.643	0.554
**[SD]**	[0.038]	[0.090]	[0.071]	[0.068]	[0.138]	[0.143]	[0.144]	[0.108]	[0.163]	[0.133]
**VAS index value (mean)**	82.3	73.0	76.5	71.9	63.9	58.7	52.0	47.5	44.5	33.6
**[SD]**	[6.5]	[13.5]	[10.3]	[9.6]	[17.8]	[18.9]	[18.0]	[13.0]	[20.0]	[15.6]

### Regression Analysis

We merged data for the surveys 2006 and 2018 and performed OLS regression with observed EQ VAS score as outcome variable and entered survey year as a dummy variable (Table 5). In the regression analysis, we adjusted for sex, age group, country of origin, duration of homelessness, degree of homelessness, health-related behaviors, LLI, and survey year. Only LLI was significantly associated to mean EQ VAS score, and there was no significant difference between the survey years 2006 and 2018. Having LLI was associated with significantly lower (−13.06) EQ VAS score (*p* < 0.0001).

**Table 5:** Ordinary Least Squares (OLS) regression on mean observed EQ VAS score adjusted for sex, age group, country of origin, duration of homelessness, degree of homelessness, health-related behaviors, limiting longstanding illness, and survey year, homeless people in 2006 and 2018, Stockholm.

	**Estimate**	**RSE**	***p*-Value**
**Intercept**	**62.901**	**5.049**	**<0.0001**
**Sex*^a^***			
Women	4.385	3.389	0.1968
**Age group*^b^***			
<35	0.863	6.532	0.8949
35–44	2.913	3.981	0.4649
45–54	1.117	3.390	0.7419
**Country of origin*^c^***			
European countries	−2.595	4.083	0.5254
Other countries outside Europe	−0.153	4.322	0.9717
**Duration of homelessness*^d^***			
<4 years	−5.457	3.721	0.1435
4–9 years	0.345	3.385	0.9189
**Degree of homelessness*^e^***			
Rough sleepers	−0.688	3.093	0.8241
**Health-related behaviors*^f^***			
Daily smoking	4.668	3.302	0.1585
Binge drinking	−3.618	3.562	0.3107
Narcotic drug use	−1.930	3.021	0.5234
**Limiting longstanding illness*^g^***			
Yes	**−13.063**	**3.214**	**<0.0001**
**Survey year*^h^***			
2018	−2.462	3.137	0.4332
**R^2^**	0.0978
***Adjusted R* ^2^**	0.0530
**RMSE**	24.42
***N***	297

*RSE, Robust standard error; RMSE, root mean square error. Statistically significant estimates are shown in bold (<0.05)*.

*Reference groups*:

a *Men*.

b *55+ years*.

c *Sweden*.

d *10 years or more*.

e *Not rough sleepers*.

f *No daily smoking, binge drinking or narcotic drug use*.

g *No limiting longstanding illness*.

h *2006*.

In the regression analysis (Table 5), females had higher mean EQ VAS score. Those in the oldest age group (55+ years) and those originating from outside Europe had lower EQ VAS score. Those with shorter duration of homelessness and rough sleepers had lower EQ VAS scores. Smokers had higher EQ VAS scores while those reporting binge drinking and using narcotic drugs had lower EQ VAS scores. Survey year 2018 was associated with lower mean EQ VAS score. However, none of these associations with co-variates was statistically significant.

## Discussion

Homeless people are a disadvantaged group with poor health, as evidenced by the findings of both surveys. The demographic composition of the homeless people interviewed in 2006 and in 2018 was similar but differed in some aspects, including a higher mean age and a larger proportion of persons originating from countries outside Europe in 2018 compared to in 2006. This may reflect the increased number of migrants coming to Sweden in the last decade. A majority had been homeless 10 years or more.

In 2018, a significantly lower proportion than in 2006 reported to use narcotic drugs (35.1 vs. 50%). The proportions with LLI and reporting less than good health were higher in 2018 than in 2006. In the 2018 survey, a greater proportion also reported to have been exposed to violence in the last year, to lack social support and to have refrained from seeking medical care and from purchasing prescribed pharmaceutical drugs, for economic reasons.

Another difference was found in the forms of subsistence: the proportion receiving some form of social insurance benefit was substantially lower in 2018 than in 2006, while the proportion receiving social assistance was higher in 2018 than in 2006. This may be important, as social insurance benefits (sickness benefit, disability pension) which are provided after medical certification in recognition of serious health problems, are more generous and in some cases (e.g., disability pension) more long-term than social assistance. Social assistance on the other hand is a temporary, means-tested, and less generous benefit, which is applied for and reviewed monthly, not specifically based on health conditions and provided by municipalities. The lower proportion having social insurance benefits in 2018 may reflect a general increased restrictiveness of the Swedish Social Insurance Agency to grant disability pension in spite of medical certification of the need for it. In 2016, some 70% of applications for disability pension were rejected, which leaves an increasing proportion “too sick to work, too healthy to qualify” (13). In the clinical setting, homeless people who were previously granted disability pension were relieved from the stress of constantly applying for social assistance, but the current restrictiveness regarding disability pension is associated with worry and anxiety among homeless people.

It is possible that some of the interviewees in our survey reflect the group referred to as “structural homeless” (16), i.e., that they are homeless only because of poverty, unemployment, and lack of affordable housing. However, while some of the interviewees stated that their problems with homelessness were mainly lack of housing, the majority of the interviewees reported other social problems, a high rate of physical and mental health problems, and narcotic drug use. Some of the interviewees were disappointed that the welfare state institutions had not intervened to help them when needed, and one stated that “the societal climate is getting tougher,” especially for those who are sick and poor. A majority had longstanding health problems, both mental and physical, some of which had contributed to their homelessness, and some of which were consequences of, or aggravated by their homelessness, as found in other studies (3).

The homeless people interviewed responded to the EQ-5D-3L questionnaire in both survey years, which enabled a comparison over time, and also comparison with the general population. Comparisons to EQ-5D-5L population reference data for Sweden, which investigates inequality and heterogeneity in health-related quality of life, shows that the average level of health of homeless people is very poor, and that among the homeless, most problems were reported with anxiety/depression, while pain/discomfort was most reported in the general population (22). The mean observed EQ VAS score among the homeless people in 2018 was 53.4, a score which is comparable to the score among persons aged 95–104 years (52.8) in the general Swedish population 2017 (22). The mean EQ VAS score in the ages 35–54 was about 52, compared to 78 in the general population in the same ages. This indicates that homeless people may have an earlier onset of disease and acquire age-related functional impairments at an earlier age than those in the general population, as observed in American studies (3). It is also similar to the observation from the United Kingdom that multimorbidity and physical-mental comorbidity is more common among people in socioeconomically deprived areas, where the onset of multimorbidity occurs 10–15 years earlier than among people in less deprived areas (23). Our findings are similar to those in a study in United Kingdom (UK) by Lewer et al. (24), and an Italian study by Leworato et al. (25), which both showed high levels of morbidity, high levels of problems in anxiety/depression, and low EQ VAS scores among homeless people (24, 25). The UK study compared homeless and those with housing, and found a “slope” in health among the general population with housing, and “a cliff” when considering homeless people, both in terms of chronic somatic conditions, infections, and mental health (24).

Comparing health among homeless people over time, a higher proportion of respondents in 2018 reported problems in different dimensions, significantly higher with pain/discomfort. The mean TTO and VAS index values were significantly lower among men in 2018 than in 2006.

When analyzing the reporting of problems in EQ-5D-3L dimensions by SRH severity level, there was a gradient with increasing reporting of problems and lower mean EQ VAS scores, with poorer SRH. This suggests that the EQ-5D-3L mirrors the commonly used single response SRH question, but EQ-5D-3L also provides information on which dimensions of health are affected.

The regression analysis comparing the health of homeless people in 2018 and 2006 adjusted for several co-variates found that having a LLI was most strongly associated to lower mean EQ VAS score in both surveys. There was no statistically significant difference between the survey years in the analysis when controlling for co-variates. The findings of the regression analysis are similar to that of Sun et al. (2).

A limitation of this study is the relatively small sample sizes in both surveys, which limits the statistical power of analysis. It is difficult to define the degree of homelessness in a single dimension, in our case it was based on the type of housing in the last night before the interview, but this may vary from night to night in this group. Another limitation is that the sample obtained excluded those who did not want to participate, who might have worse health, which would lead to an underestimation of the health disadvantage of homeless people. As there was no registration of name or identity, it is possible (but not likely) that a person participating in 2006 may have also participated in the 2018 survey, without our knowledge. However, no person interviewed in 2018 commented to have been interviewed in 2006.

The recruitment procedure used in the 2018 survey was similar (but not identical) to that used in 2006. The surveys were not done on specific weekdays, both were mainly done during autumn months but the 2018 survey went on into the first months of 2019. Some of the shelters visited in 2006 had closed down and new institutions had opened in 2018. However, the target group for the shelters and institutions was the same in both years, i.e., homeless people. Nevertheless, the sample of homeless people in the 2018 survey and in the 2006 survey was considered representative of the population of homeless people referred to as “acute homeless” (rough sleepers, people sleeping in shelters, emergency housing, and protected housing).

A strength of this study was that it provides an assessment of health in a disadvantaged group which is rarely included in population surveys, and are generally hard to reach. One interviewer performed the face-to-face interviews each survey year, using the same questionnaire in both surveys. Another strength is the use of a modified public health questionnaire, including the EQ-5D-3L instrument, which enables comparison also with larger population groups.

To the best of our knowledge there are no studies investigating trends or changes over time in the health of homeless people 2006–2018. This study provides information for policy makers responsible for the health and well-being of the group of homeless people. The European Federation of National Organizations Working with the Homeless (FEANTSA), aiming to end homelessness in Europe, regularly monitors progress toward this goal in European countries (26). In their latest country update in November 2020, the organization noted that Sweden has no up to date strategy on homelessness (27).

From the results of this study, including accounts by homeless people in the 2018 survey, efforts are needed to facilitate housing solutions, but also more engagement and collaboration by public authorities with responsibilities for homeless people's health and social welfare. Further studies should evaluate the impact of intensified joint efforts by health and social care services on the health and well-being of homeless people.

## Conclusions

Homeless people are an extremely disadvantaged group, also in Sweden with its comparatively generous welfare state services. Homeless people have high rates of illness and disease and report poor health in all EQ-5D-3L dimensions. The mean observed EQ VAS score among the homeless people in 2018 (53.4) is comparable to the score among persons aged 95–104 years (52.8) in the general Swedish population 2017. This indicates that homeless people may have an earlier onset of disease and acquire age-related functional impairments at an earlier age than those in the general population. They have an increased need of mental and physical health care, as well as long-term income support for their subsistence. Homeless people are hard to reach and seldom included in public health surveys. The EQ-5D-3L instrument was easily administered to this group, its use allows comparison with larger population groups. The change in the composition of homeless people over time, and changes in their health, partly reflects changes in society affecting this vulnerable group. Efforts are needed regarding housing, but also intensified collaboration by public authorities with responsibilities for homeless people's health and social welfare. Further studies should evaluate the impact of such efforts by health and social care services on the health and well-being of homeless people.

## Data Availability Statement

The datasets presented in this article are not readily available because data sharing is not possible as in the ethics applications and in the information to the interviewees it was stated that the data material would be available only to the research group.

## Ethics Statement

The studies involving human participants were reviewed and approved by the Regional Ethical Review Board, Stockholm, Sweden (Dnr: 2006/5:7; 2018/1923-31). Written informed consent for participation was not required for this study in accordance with the national legislation and the institutional requirements.

## Author Contributions

BB and KB conceived this study, performed preliminary data analysis, wrote the first draft of the manuscript. RI performed data collection in the 2006 survey and revised the work critically for important intellectual content. BB acts as the overall guarantor. All authors contributed to the study design, performed material preparation, and interpreted the data. The content of this manuscript is not under consideration for publication elsewhere and all authors have read and approved the submitted version of the manuscript, and therefore share collective responsibility and accountability for the manuscript.

## Funding

The data collections were performed within and funded by Region Stockholm. Region Stockholm did not have any influence regarding the study design, interpretation of results, or in formulating the manuscript.

## Conflict of Interest

BB is employed by Region Stockholm, which also funded the study. KB is a member of the EuroQol Group. Neither of these had any influence regarding the study design, interpretation of results or in formulating the manuscript. The remaining author declares that the research was conducted in the absence of any commercial or financial relationships that could be construed as a potential conflict of interest.

## Publisher's Note

All claims expressed in this article are solely those of the authors and do not necessarily represent those of their affiliated organizations, or those of the publisher, the editors and the reviewers. Any product that may be evaluated in this article, or claim that may be made by its manufacturer, is not guaranteed or endorsed by the publisher.
