# Supplementary material for: EQ-5D-3L Health Status Among Homeless People in Stockholm, Sweden, 2006 and 2018
Source: Front Public Health. 2021 Dec 20;9:780753. doi: 10.3389/fpubh.2021.780753 (PMC8720753; doi:10.3389/fpubh.2021.780753)
Supplement: Supplementary file 1 [file Data_Sheet_1.PDF]

Supplementary Material

1 Supplementary Table

Number and percentage of respondents by reported EQ-5D-Y-3L health state, homeless people, Stockholm

Survey year 2006 (36 health states reported)

Health states (2006)	n	%	Cumulative %
**11111**	22	14.8	14.8
**11112**	26	17.5	32.2
**11113**	6	4.0	36.2
**11121**	7	4.7	40.9
**11122**	16	10.7	51.7
**11123**	7	4.7	56.4
**11131**	3	2.0	58.4
**11132**	5	3.4	61.7
**11133**	3	2.0	63.8
**11212**	6	4.0	67.8
**11213**	1	0.7	68.5
**11221**	1	0.7	69.1
**11222**	2	1.3	70.5
**11223**	4	2.7	73.2
**12223**	1	0.7	73.8
**21111**	1	0.7	74.5
**21112**	1	0.7	75.2
**21121**	4	2.7	77.9
**21122**	7	4.7	82.6
**21123**	5	3.4	85.9
**21132**	1	0.7	86.6
**21133**	1	0.7	87.3
**21212**	1	0.7	87.9
**21222**	1	0.7	88.6
**21223**	1	0.7	89.3
**21232**	1	0.7	89.9

**21233**	3	2.0	92.0
**21331**	1	0.7	92.6
**21333**	2	1.3	94.0
**22122**	1	0.7	94.6
**22123**	1	0.7	95.3
**22222**	2	1.3	96.6
**22231**	1	0.7	97.3
**22233**	2	1.3	98.7
**22333**	1	0.7	99.3
**32123**	1	0.7	100.0

Survey year 2018 (48 health states reported)

**Health states (2018)**	**n**	**%**	**Cumulative %**
**11111**	8	5.4	5.4
**11112**	9	6.1	11.5
**11113**	5	3.4	14.9
**11121**	5	3.4	18.2
**11122**	15	10.1	28.4
**11123**	11	7.4	35.8
**11131**	4	2.7	38.5
**11132**	4	2.7	41.2
**11133**	4	2.7	43.9
**11212**	4	2.7	46.6
**11213**	1	0.7	47.3
**11221**	2	1.4	48.7
**11222**	3	2.0	50.7
**11223**	6	4.1	54.7
**11233**	2	1.4	56.1
**11313**	1	0.7	56.8
**11323**	1	0.7	57.4
**11332**	1	0.7	58.1
**11333**	1	0.7	58.8
**12122**	1	0.7	59.5

**12222**	1	0.7	60.1
**12223**	2	1.4	61.5
**21111**	1	0.7	62.2
**21112**	1	0.7	62.8
**21121**	3	2.0	64.9
**21122**	7	4.7	69.6
**21123**	2	1.4	71.0
**21131**	1	0.7	71.6
**21132**	5	3.4	75.0
**21133**	2	1.4	76.4
**21212**	1	0.7	77.0
**21221**	1	0.7	77.7
**21222**	3	2.0	79.7
**21223**	4	2.7	82.4
**21232**	6	4.1	86.5
**21233**	8	5.4	91.9
**21323**	1	0.7	92.6
**21331**	1	0.7	93.2
**21333**	1	0.7	93.9
**22121**	1	0.7	94.6
**22132**	1	0.7	95.3
**22211**	1	0.7	96.0
**22221**	1	0.7	96.6
**22323**	1	0.7	97.3
**22333**	1	0.7	98.0
**23321**	1	0.7	98.7
**32332**	1	0.7	99.3
**33333**	1	0.7	100.0
